# Perceived stigma, common mental disorders and associated factors among primary caregivers of adult patients with mental illness attending at public hospitals, Harari regional state, Eastern Ethiopia: A multicenter cross-sectional study

**DOI:** 10.3389/fpubh.2023.1024228

**Published:** 2023-03-02

**Authors:** Kabtamu Nigussie, Dejene Tesfaye, Tilahun Bete, Henock Asfaw

**Affiliations:** Department of Psychiatry, School of Nursing and Midwifery, College of Health and Medical Science, Haramaya University, Harar, Ethiopia

**Keywords:** magnitude, caregivers, associated factors, perceived stigma, common mental disorders

## Abstract

**Background:**

Giving care to someone who is experiencing mental distress is a difficult and challenging task that could be detrimental to the caregiver's overall quality of life. Numerous studies show that caregivers frequently experience mental distress and feel stigmatized. However, there is limited study done on this topic in our country.

**Objective:**

To determine the magnitude and associated factors of perceived stigma and common mental disorder among primary caregivers of adults with mental illness in public hospitals at Harari regional state, Eastern Ethiopia, 2022.

**Methods:**

A cross-sectional study design was conducted. A total of 419 participants were recruited by a systematic random sampling technique. The data was collected by using a face-to-face interview and by reviewing patients' charts. Perceived stigma and common mental disorder were assessed by Family Interview Schedule and Self-Reporting Questionnaire, respectively. Epi-data version 4.6.2 was used to enter data, and SPSS version 20 was used to analyze. Bivariable and multivariable logistic regression were performed. *P*-values < 0.05 were considered statistically significant.

**Result:**

The magnitude of perceived stigma and common mental disorder were 42.5% (95% CI, 37.7–47.3) and 39.4% (95% CI, 34.8–44.0) respectively. Age of caregiver between 26 and 33 [AOR = 3.13, 95% CI: (1.71–8.93)], no formal education [AOR = 3.85, 95% CI: (1.81–8.15)], illness duration ≥7 years [AOR = 1.93, 95% CI: (1.04–3.57)], family history of mental illness [AOR = 1.92, 95% CI: (1.09–3.39)] and poor social support [AOR = 4.87, 95% CI: (3.74–12.71)] were significantly associated with perceived stigma. Being female [AOR = 1.92, 95% CI: (1.31–3.34)], having no formal education [AOR = 4.04, 95% CI: (2.15–10.01)], having a family history of mental illness [AOR = 2.26, 95% CI: (1.29–4.00)], having comorbid other illness [AOR = 2.13, 95% CI: (1.15–3.94)], and having poor social support [AOR = 4.58, 95% CI: (2.53–8.28)] were significantly associated with common mental disorder.

**Conclusion:**

The magnitude of perceived stigma and common mental disorder was high among primary caregivers of patients with mental illness compared with other studies. Age of caregiver between 26 and 33, no formal education, duration of illness ≥7 years, family history of mental illness and poor social support were associated with perceived stigma and being female, no formal education, family history of mental illness, comorbid other illness and poor social support were associated with common mental disorder.

## Introduction

The most frequent and challenging issues in daily life are mental disorders, which are typically characterized by a combination of abnormal thoughts, perceptions, emotions, behavior, and interactions with others. They include: depression, bipolar disorder, schizophrenia and other psychoses, dementia, and developmental disorders. Their magnitude indicates that over 25% of people experience a mental disorder at some point in their lifespan (1–3).

A common mental disorder (CMD) is one of the most common mental illnesses in the world, and it refers to a set of illnesses that commonly include depression, anxiety, and somatoform disorders (4). A mental disorder is characterized by a clinically significant disturbance in an individual's cognition, emotional regulation, or behavior. It is usually associated with distress or impairment in important areas of functioning. There are many different types of mental disorders. In most cases, they do not impair insight or cognition, but they do cause significant emotional pain and interfere with daily activities. The cumulative cost of CMDs to society is substantial despite the fact that they are typically less impairing than serious psychiatric diseases due to their higher prevalence (5).

Stigma is a social process that is characterized by separation, rejection, and blame or discrediting of an individual or group (6). It takes place on three levels: organizational, public, and individual. The stigma that exists at the individual level, or perceived stigma, refers to the respondent's perceptions that people with mental illness are typically stigmatized (7).

Caregivers are individuals who pay attention of others, often parents, spouses, or kids with special medical needs or disabilities (8). Caregiving is a broad responsibility that involves not only providing physical assistance and care but also emotional support. It is frequently a very stressful task that creates difficulties for the caregivers on the social, emotional, behavioral, and financial aspects, making them prone to common mental disorders like depression, anxiety, and somatic problems (9, 10).

The most prevalent cause of disability worldwide is mental illness, which includes common mental disorders, neurological disorders, substance use disorders, and suicide. Over 450 million people are thought to be affected by mental disorders internationally (11). CMD has recently been found to be 14% of the overall illness burden. It is anticipated that by 2030, it will rank as the primary cause of disease burden (12).

Studies have shown that family caregivers of patients with mental illness frequently receive insufficient support from mental health specialists and experience common mental disorder themselves. According to research from Latin America, 40% of caregivers met the criteria for being at risk of depression, compared to 13–18% of the general population (13). Nearly half (43.8%) of caregivers of psychiatric outpatients in Nigerian research experienced psychological distress (14).

One of the most difficult psychosocial challenges that family members or caregivers of people with mental illness must face is stigma. Evidence from around the world shows that the psychosocial burden on families of those with mental illnesses negatively affects both the family members and the patients they are caring for (15–17). One Moroccan study on family members of schizophrenia patients revealed significant levels of perceived stigma and burden on their families (18).

Among primary caregivers of patients with mental illness, a number of factors were consistently associated to common mental disorder, including the length and type of care provided, unemployment, the length of the illness, behavioral problems, cognitive and functional disabilities, feelings of isolation, anger, sadness, and guilt, poor social support, patient age, negative coping mechanisms, and real stigma (13, 14, 19).

Despite the fact that several studies have been conducted in the United States (US), Europe, and Africa. However, there have been few studies conducted in Ethiopia, and no study has been conducted in eastern Ethiopia regarding the magnitude of common mental disorders and perceived stigma among caregivers of patients with mental illness in Harari Regional State, eastern Ethiopia. Therefore, the purpose of this study is to assess the magnitude of perceived stigma, common mental disorders and associated factors among primary caregivers of adult patients with mental illness attending public hospitals, Harari Regional State, eastern Ethiopia.

## Methods and materials

### Study area and period

Public hospitals in the Harari Regional State were used to perform the study. Harari Regional State is one of the 11 regions in Ethiopia, located in the eastern part of the country. The districts of Oromia regional state that form the borders of the region are Fedis district in the south, Gursum and Babile districts in the east, Haramaya district including Aweday in the west, and Kombolcha and Jarso districts in the north., which are the districts of Oromia regional state. The Harari Region is divided into six urban and three rural administrative districts in accordance with the present administrative system. The region had 183 344 people overall, 99 321 (54.2%) of whom lived in cities, and 84 023 (45.8%) of whom lived in rural areas. According to the Harari regional health bureau spokesperson, there are two public, one police, and two private hospitals. There are also eight (four urban and four rural) health centers, 19 health posts and 10 private clinics in the region. The study was conducted from April 01 to April 30, 2022.

### Study design and population

An institutional-based quantitative cross-sectional study design was employed. All the primary caregivers of adult patients with mental illness attending public hospitals in Harari regional state, eastern Ethiopia who were available during the data collection period and whose age was ≥18 years were included in the study. Caregivers who were unable to communicate due to the severity of their health conditions were excluded.

### Sample size determination and sampling procedure

The sample size for this study was calculated using the formula for estimation of single population proportion by considering the proportion of outcome variables of perceived stigma among primary caregivers studied in Jimma City was 54.7% (20), and Z value corresponding with a 95% confidence level (1.96); and d is the 5% desired precision.

Where:


$$\begin{array}{lll} n_{i}\; & = & \;\frac{{\left(Z\alpha 1{/}_{2}\right)}^{2}p\left(1-p\right)}{d^{2}}\\ n_{i} & = & \frac{{\left(1.96\right)}^{2}\times \left(0.547\right)\times \left(1-0.547\right)}{{\left(0.05\right)}^{2}}=381 \end{array}$$

Including 10% of the non-response rate, the final sample size was 419.

**Figure 1:**
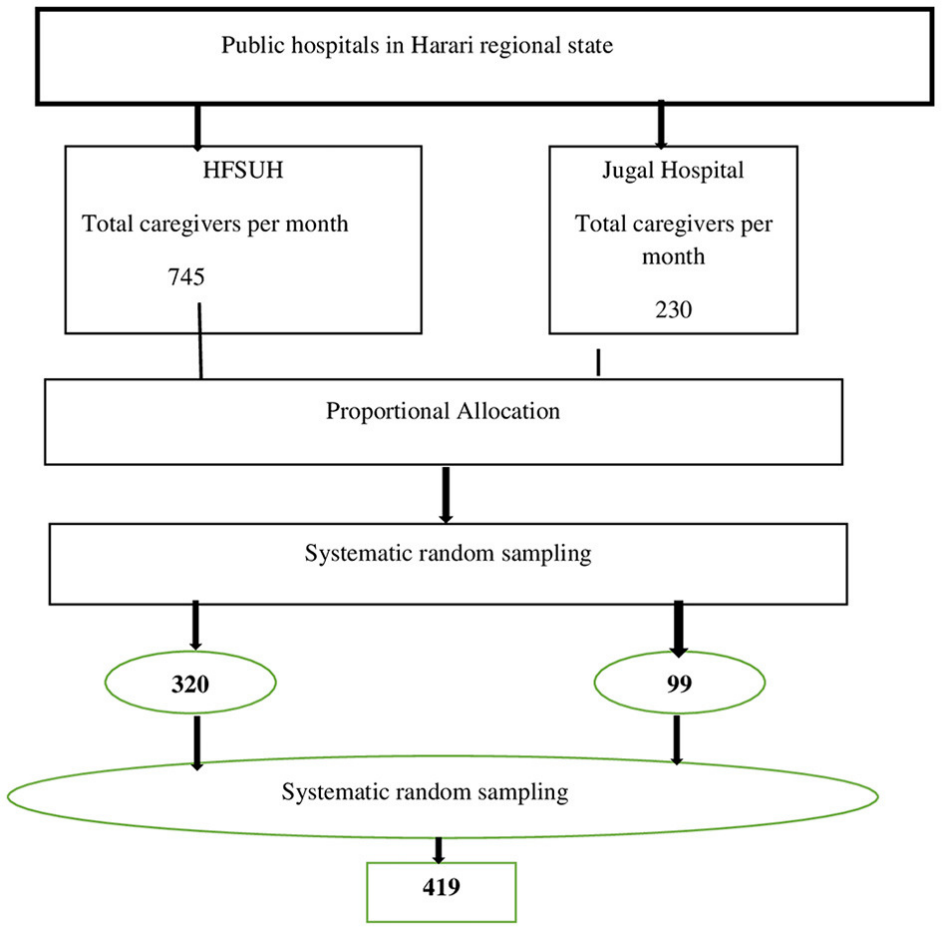
The schematic presentation of the sampling procedure for the study on magnitude of perceived stigma and CMD and associated factors among caregivers of patients with mental illness attending public hospitals of Harari regional state 2022.

Concerning the sampling procedure, the estimated sample was proportionally allocated to each hospital based on the number/load of patients attending psychiatric units. Finally, a systematic random sampling technique was used to reach the actual primary care givers. The total number of patients attending psychiatric services at the psychiatric unit was 900 per month at HFSUH and 300 at Jugal hospital. Among these patients, 745 and 230 of the patients come with their care givers to HFSUH and Jugal, respectively. Then the total number of primary care givers was divided by the simple size and the interval (K = 2) was established. Then, based on the calculated K-value, the data was collected from both hospitals. Therefore, the sampling interval was 2. The first study participant was selected by a lottery method from each hospital independently, and the next study participants were chosen at a regular interval (every 2). They were interviewed by data collectors as shown in Figure 1.

### Data collection tool and procedure

An interview-based, pretested, and semi-structured questionnaire adapted from different literatures was administered. A face-to-face interview technique was used to collect the data. The questionnaire solicits information about the socio-demographic conditions, COVID-19 related variables, mental health problems and behavioral variables, the presence of suicidal behaviors, medication non-adherence, sleep disturbances, and quality of life among patients having mental illness.

#### Self-reporting questionnaire (SRQ-20)

An SRQ-20 that had been locally validated was used to measure CMD. The SRQ-20 consists of 20 yes/no questions on whether you have ever experienced depressive, anxiety, panic, or somatic symptoms in the preceding 30 days. The total score was dichotomized and score < 6 was regarded as no CMD and score greater or equal to 6 was regarded as having CMD, high scores indicating a high level of CMD. The tool was developed by World Health Organization and assesses primarily non-psychotic mental illness. It has 20 questions, answered by YES or NO with codes “1” represents the presence of a symptom, and “0” if the symptom is absent (21).

#### Current substance use

The WHO Alcohol, Smoking, and Substance Involvement Screening Test (ASSIST) test was used to evaluate the substance use behavior of primary care providers of patients with mental illness (22).

Social support was assessed using the Oslo 3-item Social Support Scale. This test measures the degree of social support in a very quick and cost-effective manner. A score of 3–8 represents poor social support, a score of 9–11 represents moderate support, and 12–14 represents strong support in this study (23).

The brief resilient coping scale (BRCS-4) was used to assess the coping strategies for difficult situations. Those primary care givers who scored 4–13 points were considered low-resilient coopers, 14–16 points were medium-resilient coopers, and 17–20 points were highly resilient coopers (24).

Family Interview Schedule: this is an instrument developed by WHO as part of the study on the course and outcome of schizophrenia. The stigma component of this instrument was used in different parts of Ethiopia; Jimma (20, 25), and Bahir Dare (26). The stigma part of this instrument has 14 items which can be rated on a four-level Likert scale of 0 means “not at all,” 1 means “sometimes,” 2 means “often” and 3 means “a lot.” In the 14 items, the sum of all scores ≥1 was computed. Those who scored below the mean were considered as “low stigma,” and those who scored above the mean were considered as “high stigma” (27). Data were collected by three trained BSc psychiatric nurses and supervised by one MSc in mental health professional. COVID-19 prevention protocol was completely applied during data collection.

### Data quality control

The questionnaire was initially prepared in English, translated by experts into Afaan Oromo and Amharic, and then returned to English in order to maintain the consistency of the data. To ensure the quality of the data, we adapted standardized and validated tools specific to the research objectives. The supervisor and data collectors received intensive 1-day training. One week prior to the actual data collection a pre-test was conducted among 5% of the sample size in Jinela Health Center In order to evaluate the acceptability and applicability of the procedures and tools. The supervisors and investigators constantly monitored data collectors throughout the data collection process to maintain the consistency and completeness of the questionnaire. In order to reduce errors during data input, double data entry was done by two data clerks.

### Data processing and analysis

The gathered information was entered into EPI Data version 3.1, and SPSS version 20 was used for analysis. In order to describe categorical variables, frequency and percentage were used. After being evaluated for normality, continuous variables were described using the relevant summary measures and Bi-variable and multivariable binary logistic regression were carried out. The relationship between the dependent and independent variables was examined.

To identify significantly associated variables in the model, independent variables from bivariable logistic analysis with a *p* < 0.25 were fitted into multivariable logistic regression. The strength of the association was determined using the odds ratio with the 95% confidence interval, and association to be statistically significant if the *p*-value was < 0.05 at final model. In all of the models, the multi-collinearity of independent variables was tested using the variance inflation factor (VIF). In addition, model-goodness of fit was checked by using the Hosmer-Lemeshow test.

## Results

### Socio-demographic characteristics of the respondents

From a total of 419 samples, 414 participants were included in the study, with a response rate of 98.8%. Regarding the sex of both patient and caregiver, the majority of patients [231 (55.8%)] were male. However, both sexes equally participated regarding caregiving. The result of the age of both patients and caregivers showed that 113 (27.3%) patients were in the age range of 26–33 years and 120 (29%) of caregivers were in the age range of 34–41 years. Regarding the marital status and religion, about 186 (44.9%) of participants were married, and 184 (44.4%) were Islamic followers. Occupation and educational status were also studied variables, and the result showed that 113 (27.3%) were farmers and 173 (41.8%) had no formal education. Regarding the residence of participants, 195 (52.9%) came from urban areas and 107 (25.8%) earned a monthly income of < 1,000 ETB as shown in Table 1.

**Table 1:** Socio-economic distributions among primary caregivers of adult patients with mental illness attending at Harari region state public hospitals, Eastern Ethiopia, 2022 (*n* = 414).

**Variables**	**Categories**	**Frequency (*n* = 414)**	**Percentage (%)**
Sex of patient	Male	231	55.8
	Female	183	44.2
Sex of caregiver	Male	204	49.3
	Female	210	50.7
Age of patient in year	18–25	84	20.3
	26–33	113	27.3
	34–41	111	26.8
	42–49	43	10.4
	≥50	63	15.2
Age caregivers in year	≤ 25	79	19.1
	26–33	79	19.1
	34–41	120	29.0
	42–49	82	19.8
	≥50	54	13.0
Marital status	Married	186	44.9
	Single	88	21.3
	Divorced	76	18.4
	Widowed	64	15.5
Religion	Muslim	184	44.4
	Orthodox	143	34.5
	Others^*^	87	21.1
Occupation	Governments	49	11.8
	Private business	86	20.8
	Farmer	113	27.3
	Students	79	19.1
	Household wife	51	12.3
	Unemployed	36	8.7
Education	No formal education	173	41.8
	Primary school (1–8)	109	26.3
	Secondary school (9–12)	68	16.4
	College and above	64	15.5
Place of residence	Urban	195	52.9
	Rural	219	47.1
Average monthly income in EBT	≤ 1,000	107	25.8
	1,001–2,500	88	21.3
	2,501–5,000	84	20.3
	5,001–7,500	70	16.9
	≥7,501	65	15.7

^*^Others = protestants, catholic and Wakefata.

### Clinical, substance use, and psychosocial characteristics of respondents

Regarding the clinical characteristics of the respondents, about 85 (20.5%) had a family history of mental illness, 157 (37.9%) had a patient whose illness lasted for < 1 year, 153 (37%) spent the whole day giving care to the patient, 196 (47.3%) had a patient whose illness was a second episode, 109 (25.8%) had a comorbid illness, 123 (29.8%) had a patient who was not admitted to hospital, 123 (29.8%) had a patient who was diagnosed with schizophrenia, and 118 (28.5%) were given care for < 1 month. Regarding substance use, 193 (46.6%) had used any substance in their lifetime and 149 (36%) were current users. Regarding psychosocial factors, 159 (38.4%) had moderate social support and 128 (30.9%) had poor social support as shown in Table 2.

**Table 2:** Clinical, substance use, psychosocial characteristics among primary caregivers of adult patients with mental illness attending at Harari region state public hospitals, Eastern Ethiopia, 2022 (*n* = 414).

**Variables**	**Categories**	**Frequency (*n* = 414)**	**Percentage**
Family history of mental illness	Yes	85	20.5
	No	329	79.5
Duration of illness	Up to 1 years	157	37.9
	1–3 years	82	19.8
	4–6 years	79	19.1
	≥7years	96	23.2
Patients suicidal attempt	No	306	73.9
	Yes	108	26.1
Hours spent with patient in a day	1–6 h	119	28.7
	7–12 h	142	34.3
	Whole day	153	37.0
Number of episode of patients illness	First	62	15.0
	Second	196	47.3
	Recurrent	156	37.7
Comorbid other illness	No	313	74.2
	Yes	109	25.8
Number of admission	First time	87	21.0
	Second time	103	24.9
	Multiple	101	24.4
	No admission	123	29.8
Diagnosis of patients	Schizophrenia	123	29.7
	Bipolar disorders	97	23.4
	Major depressive disorder	106	25.6
	^*^Others	88	21.2
Duration of care giving	Up to 1 month	118	28.5
	1 month−1 year	108	26.1
	2–3 years	106	25.6
	≥4 years	82	19.8
Ever substance use	Yes	193	46.6
	No	221	53.6
Current substance use	Yes	149	36.0
	No	265	64.0
Social support	Poor	128	30.9
	Moderate	159	38.4
	Strong	127	30.7

^*^Others = substance related disorders, anxiety disorders and other psychosis.

### Magnitude of perceived stigma and common mental disorder

Magnitude of perceived stigma and common mental disorder among participants were 42.5% (95% CI, 37.7–47.3) and 39.4% (95% CI, 34.8–44.0), respectively.

### Factors associated with perceived stigma

In bivariable logistic analysis, variables; age of caregivers, educational status, number of admission, duration of illness, time spent with patient in day, family history of mental illness and social support were found to have a *p*-value < 0.25. These variables fulfilled minimum requirements for further multivariable logistic regression model.

From multivariable logistic regression variables; age of caregiver between 26 and 33, no formal education, duration of illness ≥7 years, family history of mental illness and poor social support were statistically significant with perceived stigma at *p*-value < 0.05.

In this study, the odds of perceived stigma among participants whose ages were between 26 and 33 years old was about 3.13 times higher as compared with age ≥50 years [AOR = 3.13, 95% CI: (1.71–8.93)].

The results of this study showed that the odds of perceived stigma among participants with no formal education was about 3.85 times higher as compared with educational status of college or above [AOR = 3.85, 95% CI: (1.81–8.15)].

The odds of perceived stigma among participants whose duration of illness was ≥7 years was about 1.93 times higher as compared with participants whose duration of illness was < 1 year [AOR = 1.93, 95% CI: (1.04–3.57)].

In this study, the odds of perceived stigma among participants with a family history of mental illness were 1.92 times higher as compared with participants without family history of mental illness [AOR = 1.92, 95% CI: (1.09–3.39)].

Social support was also another factor which was associated with perceived stigma. Participants with poor social support were 4.87 times more likely to perceive stigma as compared with participants with strong social support [AOR = 4.87, 95% CI: (3.74–12.71)] as shown in Table 3.

**Table 3:** Factors associated with perceived stigma in multivariate and bivariate logistic regression analysis among primary caregivers of adult patients with mental illness attending at Harari region state public hospitals, Eastern Ethiopia, 2022 (*n* = 414).

**Explanatory variables**	**Perceived stigma**		**COR (95% CI)**	**AOR (95% CI)**
	**High**	**Low**		
**Age of caregiver in year**				
≤ 25	33	46	2.26 (1.05–4.83)	2.02 (0.84–4.89)
26–33	41	38	3.40 (1.59–7.31)	**3.13 (1.71–8.93)** ^**^
34–41	56	64	2.76 (1.34–5.63)	2.11 (0.37–7.06)
42–49	33	41	2.12 (0.99–4.56)	1.99 (0.84–4.74)
≥50	13	41	1	1
**Educational status**				
No formal education	91	82	3.63 (1.89–6.95)	**3.85 (1.81–8.15)** ^**^
Primary school	47	62	2.48 (1.24–4.95)	2.16 (0.98–4.78)
Secondary school	23	45	1.67 (0.78–3.59)	1.54 (0.71–4.25)
College and above	15	49	1	1
**Number of admission**				
One times	26	61	0.56 (0.32–1.01)	1.48 (0.25–1.94)
Two times	36	67	0.71 (0.41–1.22)	0.58 (0.31–1.07)
Multiple	61	40	2.01 (1.18–3.44)	1.12 (0.58–2.10)
No admission	53	70	1	1
**Duration of illness**				
Up to 1 year	57	100	1	**1**
1–3 years	29	53	0.96 (0.55–1.68)	0.99 (0.52–1.91)
4–6 years	38	41	1.63 (0.94–2.82)	1.78 (0.92–3.44)
≥7 years	52	44	2.07 (1.24–3.48)	**1.93 (1.04–3.57)** ^*^
**Time spent with patient in day**				
1–6 h	39	80	1	1
7–12 h	61	81	1.54 (0.93–2.56)	1.66 (0.89–3.07)
Whole day	76	77	2.02 (1.23–3.23)	2.07 (0.16–3.56)
**Family history of mental illness**				
Yes	49	36	2.17 (1.33–3.51)	**1.92 (1.09–3.39)** ^*^
No	127	202	1	**1**
**Social support**				
Poor	86	42	5.38 (3.15–9.20)	**4.87 (3.74–12.71)** ^**^
Moderate	55	104	1.39 (0.84–2.31)	1.73 (0.98–3.06)
Strong	35	92	1	1

^*^p < 0.05, ^**^p < 0.001, Chi square = 5.80; DF = 8 and Hosmer-Lemshow test = 0.73. Bold values showed significant association.

### Factors associated with common mental disorder

In bivariable logistic analysis, variables such as sex, marital status, educational status, family history of mental illness, duration of illness, comorbid other illness, and social support were found to have a *p*-value < 0.25. These variables fulfilled minimum requirements for further multivariable logistic regression model.

Being female, having no formal education, having a family history of mental illness, having comorbid illness, and having poor social support were statistically significant with common mental disorder at a *p*-value < 0.05.

Being female was associated with common mental disorder in this study. The odds of having a common mental disorder among female participants were about 1.92 times higher as compared to male participants [AOR = 1.92, 95% CI: (1.31–3.34)]. No formal education was also associated with common mental disorder in this study. The odds of having a common mental disorder among participants with no formal education was about 4.04 times higher as compared to those whose educational level is college or above [AOR = 4.04, 95% CI: (2.15–10.01)].

The results of this study also showed that the odds of having a common mental disorder among participants with a family history of mental illness was about 2.26 times higher as compared with participants without a family history of mental illness [AOR = 2.26, 95% CI: (1.29–4.00)]. The odds of having a common mental disorder participants with comorbid other illness were about 2.13 times higher as compared with participants without comorbidity [AOR = 2.13, 95% CI: (1.15–3.94)].

Social support was also another factor which was associated with common mental disorder. Participants with poor social support were 4.58 times more likely to have a common mental disorder as compared with participants with strong social support [AOR = 4.58, 95% CI: (2.53–8.28)] as shown in Table 4.

**Table 4:** Factors associated with common mental disorders in multivariate and bivariate logistic regression analysis among primary caregivers of adult patients with mental illness attending at Harari region state public hospitals, Eastern Ethiopia, 2022 (*n* = 414).

**Explanatory variables**	**Common mental disorders**		**COR (95% CI)**	**AOR (95% CI)**
	**Yes**	**No**		
**Sex**				
Male	64	140	1	**1**
Female	99	111	1.95 (1.31–2.92)	**1.92 (1.31–3.34)** ^*^
**Marital status**				
Married	62	124	1	1
Single	27	61	0.89 (0.51–1.53)	0.51 (0.23–1.45)
Divorced	35	41	1.71 (0.99–2.94)	0.73 (0.17–3.10)
Widowed	39	25	3.12 (1.74–5.61)	1.30 (0.18–9.10)
**Educational status**				
No formal education	86	87	4.28 (2.14–8.58)	**4.04 (2.15–10.01)** ^**^
Primary	45	64	3.05 (1.46–6.35)	2.28 (0.94–7.45)
Secondary	20	48	0.79 (0.48–4.08)	1.93 (0.77–4.83)
Diploma and above	12	52	1	1
**Family history of mental illness**				
Yes	47	38	2.27 (1.40–3.68)	**2.26 (1.29–4.00)** ^*^
No	116	213	1	1
**Duration of illness**				
Up to 1 year	51	106	1	1
1–3 years	25	57	0.91 (0.51–1.62)	0.65 (0.17–2.48)
4–6 years	35	44	1.65 (0.95–2.88)	0.65 (0.10–4.17)
≥7 years	22	44	2.46 (1.46–4.14)	1.52 (0.73–3.14)
**Comorbid other illness**				
Yes	46	35	2.43 (1.48–3.98)	**2.13 (1.15–3.94)** ^*^
No	117	216	1	
**Social support**				
Poor	80	48	4.74 (1.78–8.09)	**4.58 (2.53–8.28)** ^**^
Moderate	50	109	1.31 (0.78–2.19)	1.29 (0.74–2.27)
Strong	33	94	1	1

^*^p < 0.05, ^**^p < 0.001, Chi square = 5.72; DF = 8 and 0.68 = Hosmer-Lemshow test. Bold values showed significant association.

## Discussion

Caregiving for people with mental illness has a significant influence on families and is frequently accompanied by significant stress on family members, which can lead to a reduction in social activities and, over time, isolation. The most frequent impact of caring for people with serious mental diseases included behavioral disturbances, functional impairments, physical impairments, cognitive impairments, and concern that their relative may attempt suicide (28, 29). Caring itself is an independent risk factor for CMD independent of the other life stressors they have to deal with (30).

The reported magnitude of CMD among the primary caregivers in this study was 39.4 % (95% CI: 34.8–44.0). The result of this study is in line with cross-sectional studies conducted in Saudi Arabia (38.4%) among caregivers of patients with mental disorders (31) and with a study conducted in Nigeria (43.8%) (14).

However, the findings from this study revealed a lower magnitude of CMD among caregivers of patients with mental disorders when compared with studies conducted in India (80%) (32), Nigeria (79.8%) (33), and Ethiopia (56.7%) (34). This discrepancy could be attributed to a smaller sample size and being the primary caregiver for the most chronic form of mental disorder, schizophrenia, noted in the Indian and Nigerian studies, and in the Ethiopian study, where the study was conducted among caregivers of severe mental illness. Patients with severe mental disorders require a greater need for emotional support, assisting with maintenance of social and living skills, household chores and financial arrangements, supervising medication, and monitoring signs for illness and relapse prevention (35). This study revealed a higher prevalence of CMD among caregivers of patients with mental disorders when compared with another study conducted in Nigeria (36). This discrepancy could be attributed to the different tools, i.e., the Self-Reporting Questionnaire-20 was used in the current study, but the Patient Health Questionnaire, used in the Nigerian study (37).

On the other hand, this study also revealed that the magnitude of perceived stigma among the primary caregivers among patients with mental illness was 42.5% (95% CI, 37.7–47.3). The current finding was in line with the study conducted in America 43% (38) and Ethiopia 38.5% (39). However, the result of this study was lower than the studies conducted in Ethiopia, in Bahirdar 89% (26), Butajira 75% (40), Addis Ababa 62.6% (41), Jimma 54.7% (20), Nigeria 85.3% (42), Chile 90% (43), and Belgium 86% (44). The possible justification for the variation might be the tool difference used to assess the perceived stigma. For example, the Addis Ababa study used the standardized perceived devaluation and discrimination questionnaire to assess perceived stigma, whereas this study used Family Interview Schedule. The other possible reason might be that the studies conducted in Addis Ababa, Ethiopia, Belgium, and Chile were only among schizophrenic patients, whereas this study includes caregivers of all severely mentally ill patients. Caregivers of patients with schizophrenia reported significantly higher stigma than patients with bipolar disorder and recurrent depressive disorder (45). In addition to this, the Butajira, Ethiopia, and Belgium studies limit the caregivers to only family members, whereas this study includes any kind of caregiver who supports severely mentally ill patients, and the Butajira study was a community-based study, whereas this study is an institutional-based study. On the other hand, the finding of the current study was higher than that of the study done in India 21% (46). The possible reasons might be the difference in the study setting, socio-cultural backgrounds, and tool differences.

This study reported that being female had a statistically significant association with CMD that was in line with a study done in Nepal (47) and study done in Ethiopia and a review also revealed that female caregivers were more likely to suffer from mental distress than male counterparts (48). All over the world, informal care for family members with chronic conditions or disabilities including those elderly and those with mental illness provided most commonly by females (49). Providing care for family members often entails bearing emotional, physical, social, and financial burdens that make care givers suffer from more stressful life events (50).

This study revealed that those with low educational status had a statistically significant association with CMD. Studies done in the United States of America (51) and in Nepal (47) revealed that those caregivers who had low educational status were more likely to be associated with CMD than those with higher educational status. A systematic review also reported that there is a significant association between CMD and lower educational status (52). However, a study in India reported that lower mental distress was noted among primary caregivers who had lower education (32). This might be due to a significantly high number of participants with lower educational status who were included in the Indian study.

A family history of mental illness was significantly associated with the caregivers' CMD. Caregiving for family members with mental illnesses is usually undertaken on a full-time basis. This would result in intense stress and burnout among the family members. Furthermore, the family suffers from social stigma, which is associated with a high level of mental distress among the family caregivers (53). Though personal satisfaction, strengthening family ties, and saving resources could be seen as positive aspects of caring, the negative impact could be very significant, as they give up work and leisure. They feel overwhelmed and trapped, and that they suffer in silence (54).

In this study, the presence of comorbid medical illnesses had a statistically significant association with CMD. This is evidenced by the presence of comorbid medical conditions not only associated with common mental disorders but also increases the re-admission of patients with mental disorders (55) and the presence of common mental disorders was associated with the development and progression of medical conditions like heart failure (56). The presence of common mental disorders was also associated with the subsequence diagnosis of medical conditions like hypertension (57). The prevalence of common mental disorders was higher among patients with medical conditions (58).

Those caregivers who had poor social support had a statistically significant association with CMD and this fact is supported by a study conducted in Addis Ababa, Ethiopia (34). Moreover, lack of social support has a profound effect on caregivers' outcomes, and less social support corresponds to a higher symptomatology of caregivers. Provision of care is also associated with a decline in social support and increased isolation and withdrawal of caregivers (48).

In this study, the odds of experiencing perceived stigma were higher among the age group between 26 and 33 years compared to 50 years old. The possible reason might be that being sensitive to community rejection is more common than being adaptive. Caregivers who had no formal education were more likely to experience perceived stigma as compared to those who were educated to college or above. This might be because those who are educated caregivers might have a good coping mechanism to sigma and understand the role of caregivers for mentally ill patients and understand the nature of the illness.

The odds of having perceived stigma was higher among patients with a long duration of illness (more than 7 years) compared to a short duration (< 1 year). This finding was supported by a previous study carried out in Jimma and Bahirdar, Ethiopia (20, 26). The possible explanation might be that caregivers of mentally ill patients may perceive stigma when the duration of the illness increases. The burden of treating mentally ill patients will lie on the caregivers in every aspect of their lives, including economically, psychologically, and physically, especially when the illness becomes chronic or of long duration.

Respondents who had poor social support were more likely to experience perceived stigma as compared to those who had strong social support. This study is supported by studies of Ergetie et al. (26), Tesfaw et al. (41), and Chien et al. (59). The possible justification for this might be the caregivers who had mentally ill patients in the family member may ignore themselves from social activities and not able to share responsibilities and role in the community.

## Conclusion

The prevalence of CMD and perceived stigma among the primary caregivers of patients with severe mental illness were high. Being female, low educational status, family history of mental illness, presence of comorbid medical illness and poor social support were significantly associated with CMD. Low educational status, family history of mental illness, being between 26 and 33 years old, having been ill for more than 7 years and poor social support were significantly associated with the perception of stigma among primary caregivers of patients with severe mental illnesses. Therefore, attention should be given, and essential psychosocial care should be provided, to maintain the mental health of primary caregivers of individuals with mental illness.

## Limitations of the study

Since it was a cross-sectional study design, it cannot allow establishing a temporal relationship between outcome variable and independent variables. Some inquiries in the study have sensitive issues, which might leads to social desirability bias.

## Data availability statement

The original contributions presented in the study are included in the article/supplementary material, further inquiries can be directed to the corresponding authors.

## Ethics statement

The studies involving human participants were reviewed and approved by Haramaya University College of Health and Medical Sciences' Institutional Health Research Ethics Review Committee (IHRERC). The patients/participants provided their written informed consent to participate in this study.

## Author contributions

KN, DT, TB, and HA were participated in inception of idea, proposal development, data collection, analysis, and final write up. HA and KN have participated on write up of the manuscript. All authors approved the final manuscript.
